# Parasites in Horses Kept in A 2.5 Year-Round Grazing System in Nordic Conditions without Supplementary Feeding

**DOI:** 10.3390/ani9121156

**Published:** 2019-12-17

**Authors:** Eva Tydén, Anna Jansson, Sara Ringmark

**Affiliations:** 1 Department of Biomedical Sciences and Veterinary Public Health, Swedish University of Agricultural Sciences, SE-75007 Uppsala, Sweden; Eva.Tyden@slu.se; 2 Department of Anatomy, Physiology and Biochemistry, Swedish University of Agricultural Sciences, SE-75007 Uppsala, Sweden; anna.jansson@slu.se

**Keywords:** pasture, landscape preservation, cyathostomin, pyrantel, EPG, biodiversity, body condition, welfare

## Abstract

**Simple Summary:**

Grazing horses year-round may be a means to increase biodiversity. In this study, parasite occurrence was documented on a monthly basis in 1- to 3-year-old Gotlandsruss stallions grazed year-round for 2.5 years. Horses became infected by several parasites and, when needed (>200 strongyle eggs/gram feces), horses were dewormed with the anthelmintic drug pyrantel, which has low or no ecotoxic impact on soil fauna. This strategy failed to control small strongyle occurrence. Horses excreted larger amounts of small strongyle eggs during summer–autumn than during the rest of the year, and the number of excreted eggs increased year-on-year. High small strongyle egg excretion did not seem to affect the body condition of the horses. Some horses were also infested with chewing louse, but did not scratch more than unaffected horses. We found that to keep egg excretion below 200, pyrantel was not sufficient and a substance known to be toxic to dung fauna and freshwater invertebrates had to be used on some occasions.

**Abstract:**

Horse grazing can be favorable from a biological diversity perspective. This study documented the occurrence of endo- and ectoparasites and sought to reduce parasite egg excretion with the anthelmintic drug pyrantel in 12 Gotlandsruss stallions maintained in a year-round grazing system for 2.5 years. Feces samples were collected monthly and all horses were treated with pyrantel, the anthelmintic drug of choice in biological diversity preservation, at study population mean cyathostomin eggs per gram (EPG) of >200. The relationship between cyathostomin EPG and body condition was studied, as was horse behavioral response to *Bovicola equi* (chewing louse) infestation. Eggs of cyathostomins (small strongyles), *Parascaris* spp. (roundworm), *Oxyuris equi* (pinworm), *Anoplocephala perfoliata* (tapeworm), and *Gasterophilus* spp. (botfly) were detected at least once during the trial. Excretion of cyathostomin eggs was highest during summer–autumn and increased year-on-year. No relationship was found between cyathostomin EPG and body condition. Infestation with *B. equi* did not affect the number of scratching sessions compared with unaffected horses. Therefore, in this year-round grazing system, pyrantel treatment had to be complemented with moxidectin to reduce excretion of cyathostomin eggs, thus compromising biological diversity.

## 1. Introduction

There is ongoing loss of biological diversity worldwide (Intergovernmental Science-Policy Platform on Biodiversity and Ecosystem Services, 2019). A major reason for this loss in Sweden is abandonment of grasslands and forest encroachment induced by a lack of large herbivores [1]. This study was part of a 2.5-year project studying the effect of year-round grazing, without supplementary feeding, by horses on biological diversity and horse growth, health, and welfare. A recent publication from this project reports that horse grazing increases plant diversity and the presence of pollinators [2]. Year-round horse grazing, as tested in the project, can therefore be recommended from a biological diversity perspective. However, to our knowledge, there have been no long-term studies on the occurrence of endo- and ectoparasites in such a system. A strategy to control endo- and ectoparasites is needed to keep grazing horses healthy and in functioning body condition, so that challenging weather and periodic feed shortages do not affect their health and welfare. 

All grazing horses are infected with parasites. The most important parasites to control in Swedish horses are cyathostomins (small strongyles), *Strongylus vulgaris* (large strongyle), *Anoplocephala perfoliata* (tapeworm), and *Parascaris* spp. (roundworm) [3]. All these parasites are gastrointestinal nematodes transmitted by grazing infected pasture. The infection builds up on pasture when adult worms shed their eggs in horse feces. Grazing horses are then infected by ingestion of the infective stage of the parasite. Parasite infection can have a negative impact on horse health, e.g., *S. vulgaris* and *A. perfoliata* are associated with an increased risk of colic [4,5], while a high burden of *Parascaris* spp. worms can lead to rupture of the small intestine [6]. Cyathostomins can cause larval cyathostominosis, which is associated with the sudden development of large numbers of cyathostome larvae in the wall of the large intestine [7]. In addition to the above-mentioned symptoms, parasite infection can cause weight loss and reduced growth in foals [3]. Apart from pasture-borne parasites, horses can also be infested with *Oxyuris equi* (pinworm), *Gastrophilus* spp. (bot fly), and the ectoparasite *Bovicola equi* (chewing louse). Because of the obvious negative effects of many parasites, parasite monitoring is generally recommended, especially in foals and young horses (less than three years of age) as they are more susceptible to parasite infections.

The cornerstone in controlling gastrointestinal parasites has long been regular treatment with anthelmintic drugs, which belong to three major drug classes: (i) macrocyclic lactones, (ii) benzimidazoles, and (iii) tetrahydropyrimidines. However, overuse of these drugs has resulted in problems with widespread resistance to benzimidazoles among cyathostomins and more recently also resistance to pyrantel [8]. However, in Sweden there is only suspected resistance to pyrantel among cyathostomins, with treatment efficacy 92% (confidence interval 80–96%) [9,10]. The problem is equally difficult with *Parascaris* spp., with widespread resistance to macrocyclic lactones and emerging resistance to pyrantel [8,11]. Infestations of *B. equi* are treated with a wide range of systemic or topical insecticides, including pyrethroids, organophosphates, and macrocyclic lactones [3].

From a biological preservation perspective, the use of anthelmintics in grazing animals should be low or ideally zero. The ecotoxic effects of anthelmintic drugs are mainly caused by the macrocyclic lactones. These have a wide spectrum of activity against endo- and ectoparasites, which increases the potential for impacts on non-target organisms. Drugs belonging to the macrocyclic lactones are only partly metabolized by the animal, and the main route of excretion is via feces [12]. Concentrations of macrocyclic lactones in feces from treated animals are lethally toxic for dung fauna and for freshwater invertebrates [12,13]. The two other drug classes, benzimidazoles and tetrahydropyrimidines, show little ecotoxic impact on dung or soil fauna [12].

The risk of an individual animal being heavily infected with parasites is dependent on several individual factors, among which age [14], immune response [14], and grazing behavior [15] may be the most important. To our knowledge, individual variations in parasite egg counts in young horses kept in a year-round grazing system without supplementary feeding have not been studied previously. In addition, little is known about how pasture area (enclosure) affects the variation in parasite egg excretion. If pasture area is important, such knowledge could be used to identify high- and low-risk pastures. 

The present study investigated the occurrence of gastrointestinal parasites in horses kept for 2.5-years in three enclosures in a year-round grazing system without supplementary feeding. Specific objectives of the work were to (1) Evaluate the possibility of using only pyrantel (tetrahydropyrimidine) to reduce cyathostomin egg excretion by >85% at the following monthly sample, and (2) document the effect of individual animal, enclosure, and season on parasite egg counts. The relationship between parasite egg counts and body condition and the behavioral response of horses to an outbreak of *B. equi* were also studied. The hypotheses tested were that: (i) pyrantel can efficiently control parasites in a year-round grazing system and (ii) individual animal, enclosure, and season have effects that are relevant to consider in future free-range grazing systems.

## 2. Materials and Methods 

The study was approved by Uppsala animal welfare ethics committee (license number: C28/14). 

### 2.1. Project Outline

The study was carried out between May 2014 and September 2016 in Krusenberg, Uppsala, Sweden (59°44′8″ N, 17°38′58″ E). Three enclosures (13, 11, and 10 ha), consisting of approximately one-third grassland and two-thirds forest, were established. Based on prior estimates of grass production, the grassland area provided was expected to meet the energy requirements of three or four 250-kg horses per enclosure. The land had not been grazed by horses in the years before the study, but enclosures 1 and 2 had been grazed by cattle, and enclosure 3 had been used for forage production.

An ancient, endangered native horse breed, the Gotlandsruss, was used in the study because it is known as an ‘easy keeper’ and was expected to cope with a year-round free-range system without supplemental feeding at the latitude of the study site. Twelve one-year old Gotlandsruss stallions (mean body weight 185 ± 21 kg at the start) were purchased from six breeders and all breeders delivered two or three horses, with one exception (only one horse). The horses were divided into groups of four so that horses from the same breeder and same sire were spread in different groups (horses 1–4, 5–8, and 9–12 in groups A, B, and C, respectively). The study was performed according to a Latin square design, with three periods, three enclosures, and three groups of horses (Table 1). The groups were randomly (by drawing) released into the enclosures in May 2014. The groups were rotated between the enclosures in May 2015 and May 2016, i.e., each group grazed each enclosure for one growing season. One horse had to be removed after 20 months due to an injury. 

Each enclosure contained a shelter of 16 m^2^ and water was offered in automatic water troughs, located in the forest, during spring, summer, and autumn. When the temperature was below 0 °C in winter, water was offered once/day in plastic troughs. In all enclosures, even during wintertime, water was also available from streams in the forest. A salt block with trace minerals (May 2014–August 2014: Ab Hansson & Möhring, Halmstad, Sweden, content (mg/kg): zinc 300, manganese 200, copper 80, iodine 50, selenium 20 and cobalt 12. August 2014–September 2016: Standard, KNC, Netherlands, content (mg/kg): zinc 810, copper 220, iodine 100, selenium 20) was provided in all enclosures. 

Horse body condition was scored weekly (scale 1–9) according to Henneke et al. [16]. If a horse scored <4, it was moved temporarily from the study enclosure to a nearby ungrazed enclosure and kept there until it scored >4 and conditions in the original enclosure were considered good enough for maintenance of body condition. In total, four horses were removed during the first winter, for approximately three weeks each. 

All horses were weighed on a scale every other week. Horses were checked daily for health issues (such as wounds, lameness, coughing, nose flow), thermoregulatory responses (such as shivering and sweating), and abnormal behavior (such as repeated scratching, looking at the belly, unwillingness to move, or a body position indicating depression).

### 2.2. Parasite Analysis

Fresh feces samples were collected on a monthly basis. For collection of individual samples, the horses were tracked until a minimum of two horses/enclosure defecated. Egg counts for strongyles and *Parascaris* spp. were carried out for each horse by a modified centrifugation-enhanced McMaster technique using saturated NaCl solution with density 1.18 g/cm^3^, with a theoretical sensitivity of 50 eggs per gram (EPG) [17]. 

Individual larval cultures for detection of *S. vulgaris* were performed on 50 g feces mixed with an equal volume of vermiculite (Weibulls, Sweden) according to Bellaw and Nielsen [18]. Third-stage (L3) larvae were harvested after sedimentation for 12–16 h at +20 °C by the inverted Petri dish method [19]. In brief, approximately 20 mL of the fluid were collected in a 50-mL Falcon tube and centrifuged at 248× *g* for 3 min. Larvae were examined and identified under a microscope using morphological criteria, counting 100 larvae/culture when possible [20]. 

Eggs of *A. perfoliata* were isolated from 30 g feces using the modified flotation technique described by Beroza et al. [21]. The feces were mixed with 60 mL tap water, strained into four 15-mL test-tubes, and centrifuged at 1000× *g* for 10 min. The supernatant was removed and the pellet was dissolved in sugar salt solution (saturated sodium chloride solution with 50% glucose with density 1.280 g/cm^3^) to form a convex meniscus. A cover glass (18 mm × 18 mm) was placed on top and the tubes were centrifuged for 5 min at 214× *g* in a swing-out centrifuge. The samples were left for 5 min after centrifugation, and then transferred to microscope slides and microscopically examined at 40–100 × magnification.

Infection with *O. equi* was diagnosed by applying one piece of adhesive tape (7 cm × 2 cm) to the peri-anal region of each horse and then placing the tape on a glass slide and examining it under optical microscope (40 × magnification). 

Occurrence (yes or no) of *Gasterophilus* spp. (botfly) eggs or egg shells in the horse coat was recorded on a weekly basis.

### 2.3. Bovicola Equi

In April 2015 (sunny daytime conditions), the number of *B. equi* adults and eggs in a circular area (diameter 22 mm) in the center of the forelock and mane was recorded for all horses. The location and total area of hair loss were subjectively estimated by one person for all individuals. Coat length was measured at the withers and rump, using a measuring tape, and a mean of these was calculated.

In addition, a behavioral study was performed where the numbers of scratching and rubbing sessions performed within 30 min were recorded for all horses in the study and also for a control group of horses (n = 7) known not to carry *B. equi*. These horses (age 2 years n = 3, 3 years n = 2, >6 years n = 2, two Gotlandruss and five Standardbred horses, all stallions but one) were kept in a free-range system with access to shelter on two farms nearby (within 20 km) and were changing from winter to summer coat, as were the experimental horses. These horses were checked daily (general health and behavior), as were the experimental horses. A scratching session was defined as a single or repeated scratching/rubbing movement. Three random recordings (the first horse that scratched after the 30 min session) were made, on three different horses, of the time spent on one scratching session, which was found to range from 2 to 8 s. 

### 2.4. Deworming Strategies

All drug classes used were in oral formulations and approved for use in horses in Sweden [22]. The drug of choice in this study was the tetrahydropyrimidine pyrantel embonate paste (Banminth^®^ Pharmaxim), due to the resistance of cyathostomins to fenbendazole and the documented ecotoxic effect of macrocyclic lactones. The intention was to deworm all horses when mean EPG of the study population (all 12 horses) was >200, regardless of horse group. Infections with cyathostomins and *O. equi* were treated with pyrantel embonate paste at a dosage of 19 mg/kg bodyweight (6.6 mg of pyrantel base), infections with *P. equorum* with fenbendazole (Axilur^®^ Intervet) at a dosage of 7.5 mg/kg bodyweight, and infections with *A. perfoliata* with a double dose of Banminth^®^ Pharmaxim (38 mg/kg bodyweight) according to the manufacturers’ recommendations. The reduction in cyathostomin egg excretion was calculated at the next monthly sampling occasion. One of the other drugs used for deworming during the study was ivermectin (Normectin^®^ N-vet) at 0.2 mg/kg bodyweight prior to onset of the study. Moxidectin (Cydectin^®^ Orion Pharma Animal Health) at 0.4 mg/kg bodyweight was used when the reduction in cyathostomin eggs was ˂85% at the following monthly sample. 

### 2.5. Statistical Analyses

All statistical analyses were performed in Statistical Analysis Systems package 9.4 (SAS Institute Inc., Cary, NC, USA). Differences were considered significant at *p* < 0.05. The seasons were defined thus: November–February = winter, March–June = spring, July–October = summer–autumn. Each year was defined as running from March–February, i.e., the start of spring season until the end of winter season, as the experiment started in spring. To estimate differences in EPG between enclosures, seasons, and years, Poisson regression was used (procedure GLIMMIX in SAS), including the default log-link for this model, which means that the expected count of eggs in the model was log-transformed. To account for overdispersion, the residual variance was estimated separately using the random residual statement. The model included fixed effects of individual horse, year, and season, with enclosure as random effect. To analyze the effect of horse group on EPG, the same model, but without the effect of individual horse, was used. The effect of interaction between season and year was also tested.

For analysis of possible differences in EPG counts between horses temporarily removed during the first winter due to low body condition and those that remained in the project, the same model as described above was applied, using EPG counts from the first year of the study. This included fixed effects of month and body condition class (removed or retained in study enclosure), with enclosure as random effect. Values presented are least square means (LSmeans) and standard error of the mean (SEM), unless otherwise stated. 

The numbers of horses on which *Gastrophilus* spp. eggs were found in the coat were summed up per month and analyzed for differences between months using the GLM procedure, including the effect of year. Results are presented as LSmeans ± SE. 

For analysis of differences between the experimental horses and control horses in terms of number of scratching sessions, a two-sample t-test was used. A two-sample t-test was also used to compare coat, mane, and tail length between experimental horses on which *B. equi* eggs and adults were found (n = 6) and those on which no *B. equi* eggs or adults were found (n = 6). Results are presented as means ± SD.

## 3. Results

### 3.1. Parasite Occurrence

**Figure 1:**
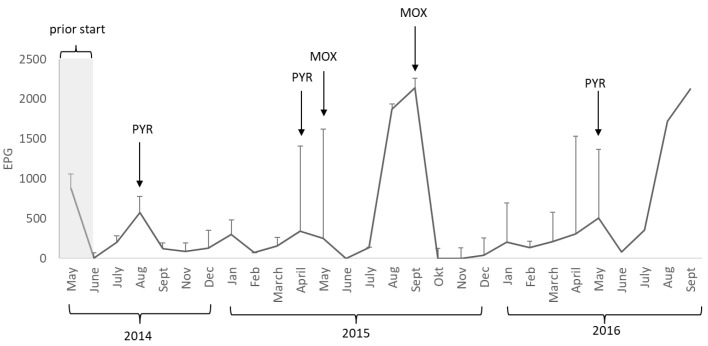
Strongyle egg excretion in eggs per gram feces (EPG). Mean of the study population (all 12 horses) (±SD) each month during 2014–2016. Arrows indicate when all horses were treated and the substance used (PYR = pyrantel, MOX = moxidectin). The egg excretion in May 2014 was before the first deworming and the onset of the study.

**Figure 2:**
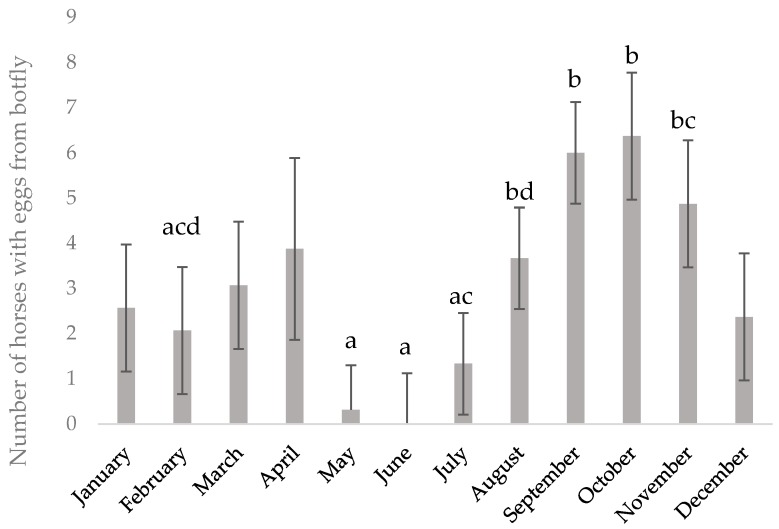
Number of horses with *Gasterophilus* spp. eggs or egg shells in each month. Results for 12 Gotlandsruss stallions maintained on year-round pasture from May 2014 to September 2016. LSmeans ± SE. Different letters (a, b, c) indicate differences (*p* < 0.05) between months.

In total, 217 feces samples were analyzed in the study, with a mean of eight samples/month. Strongyle eggs were detected in all individuals during the study (2014–2016) (Table 2). The infection level of cyathostomins gradually built up during the trial, with peak values in August/September each year (Figure 1). There was an individual effect on EPG (*p* < 0.1), with individual LSmeans for the whole study period ranging from 58 to 365 EPG (median range 25–300 EPG). All horses tested negative for *S. vulgaris* throughout the study. Egg counts of *Parascaris* spp. were detected in three of the 12 horses in January 2015, eggs of *O. equi* were detected in seven of the 12 horses in February 2015, and eggs of *A. perfoliata* were detected in five of the 12 horses in May 2015 (Table 2). In three horses (horses 2, 7, and 10), the only endo-parasite detected was strongyle eggs (Table 2). *Gasterophilus* spp. eggs or egg shells were detected in the coat of all horses at least once during the study period. The number of individuals with *Gastrophilus* spp. eggs differed between months, with the highest numbers in August–November and few eggs or egg shells found during May–July (Figure 2). No signs of discomfort that could be linked to parasites were recorded throughout the study, except for occasional tail scratching when horses were infected with *O. equi*.

### 3.2. Deworming

All horses were dewormed in May 2014 with ivermectin (Normectin^®^ N-vet) prior to division into groups (A, B, C), and released onto parasite-free pastures. During the study, all horses were dewormed when the study population mean of small strongyle EPG was >200. In August 2014 (mean EPG 564 ± 430) (Figure 1), the horses were treated with pyrantel (Banminth^®^ Pharmaxim). Egg excretion at the next monthly sampling was reduced by 78%. In April 2015, the mean EPG was 337 ± 217 and on that occasion, a double dose of pyrantel (Banminth^®^ Pharmaxim) was used because of co-infection with *A. perfoliata*. At the next monthly sample, the observed reduction in egg excretion was only 24%, which led to a second treatment in May 2015 using moxidectin (Cydectin^®^, Orion Pharma Animal Health). The observed reduction in EPG was 100% at the next monthly sample. Mean egg excretion was >200 EPG in August (1873 ± 1070) and September (2143 ± 1370) 2015, and the horses were treated with moxidectin in September, with a 100% reduction in egg excretion at the next monthly sample. In 2016, the mean EPG was 507 ± 486 in May and the horses were treated with pyrantel, with an 84% reduction in egg excretion at the next monthly sample. The EPG levels exceeded 250 (358 ± 369) in July, August (1818 ± 1205), and September (2125 ± 570) 2016, but the horses were left untreated because it was the end of the study. Despite low small strongyle EPG, two additional treatments were needed in February 2015 when some of the horses were infected with *O. equi* and *Parascaris* spp. Horses in groups A and C diagnosed with *O. equi* infections were dewormed with pyrantel (see Table 2), and horses in group B infected with *Parascaris* spp. were dewormed with fenbendazole (Axilur^®^ Intervet). In May 2016, horses that tested positive for *A. perfoliata* were dewormed with a double dose of pyrantel (Figure 1). 

### 3.3. Effects of Year, Season, Enclosure, and Horse Group

**Figure 3:**
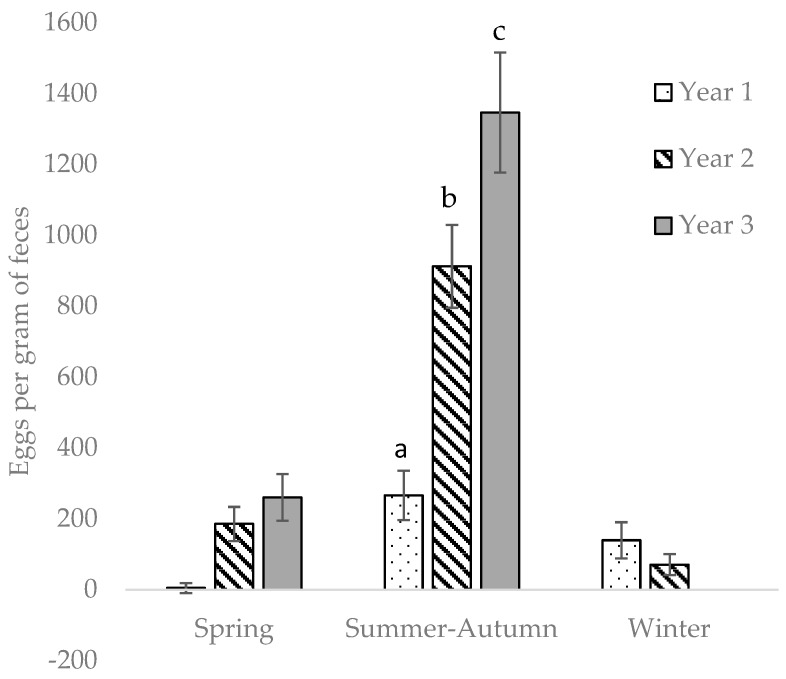
Strongyle eggs per gram (EPG) in feces samples taken monthly from 12 horses kept in a year-round grazing regime (LSmeans ± SEM). One year is defined as start of spring season until end of winter season. Different letters (a, b, c) indicate significant within-season differences between years.

Strongyle egg counts increased year-on-year (*p* < 0.01) and were higher during summer–autumn than in winter and spring (*p* < 0.01) (Table 3). For every summer–autumn season, EPG increased (Figure 3), and there was a significant interaction between season and year. There was no effect of enclosure on EPG (*p* > 0.05). Horses in group B shed higher amounts of strongyle eggs than horses in groups A and C (*p* < 0.01) (Table 3). 

The horses removed due to low body condition score had lower EPG from May 2014 to March 2015 than the horses which remained in the enclosures during the whole winter (LSmeans: 102 ± 39 vs. 211 ± 231) (*p* < 0.001).

### 3.4. Bovicola Equi

*Bovicola equi* was observed in some individuals during winter and spring in both 2014–2015 and 2015–2016, but detailed recording was performed only in spring 2015. At that time, *B. equi* adults and eggs were observed in six individuals (from all groups) and the number of eggs and live adults on affected horses ranged from 1–4 eggs in the forelock, 1–3 eggs in the mane, 1–5 lice in the forelock, and 1–2 lice in the mane. The total number of eggs plus adults counted was 1, 15, and 8 in group A, B, and C horses, respectively. The area with hair loss in affected animals was estimated to be 2.0 ± 1.7 (range 0.4–4.5) dm^2^ and the most commonly affected areas were the head, neck, and upper hind legs. The individuals affected by *B. equi* did not have different coat lengths (2.9 ± 0.4 cm) to unaffected individuals (3.1 ± 0.3 cm, *p* > 0.05), mane length (affected 38 ± 6 vs. unaffected 39 ± 5 cm), or tail length (affected 106 ± 4 vs. unaffected 107 ± 4 cm). There were no differences in number of scratching sessions between affected experimental horses (1.2 ± 1.5) and unaffected experimental horses (1.5 ± 1.9) or control horses (1.7 ± 1.8, *p* > 0.05).

## 4. Discussion

In this study, the horses started grazing clean pastures and were pre-treated with ivermectin. However, after only three months of grazing, the mean EPG for cyathostomins in the study population exceeded the threshold of 200 EPG. In addition, the horses were found to be infested with *Parascaris* spp., *A. perfoliata*, *O. equi*, *B. equi*, and *Gastrophilus* spp. (at least eggs or shells attached to the hair coat) throughout the study, but not with *S. vulgaris*. This proves the importance of monitoring parasite occurrence in young horses, even if a parasite-free pasture is used. 

It is known that encysted cyathostomins undergo arrested development in the northern temperate climate zone [23,24], and that these encysted mucosal stages are generally not susceptible to single-dose anthelmintics (except moxidectin) [25]. Parasites in this stage were not affected by the ivermectin treatment performed prior to onset of our study and were the source of contamination of the pastures. 

The objective in this study was to use pyrantel for treatment of endoparasites, due to the widespread resistance to benzimidazoles reported in cyathostomins [8] and the documented ecotoxic effect of macrocyclic lactones [13]. Pyrantel-only treatment was not successful in reducing cyathostomin egg excretion by 85%. After two treatments with pyrantel (in August 2014 and April 2015), the observed reduction in egg excretion was only 24% at the next monthly sample. This required deworming with moxidectin in May 2015, with a reduction in egg excretion of 100% at the next monthly sample, but cyathostomin eggs re-occurred after only eight weeks, indicating a shorter egg reappearance period (ERP) than expected [5]. As the mean EPG in the study population was escalating in August (1875 ± 1070) and September (2143 ± 1370) 2015, a second treatment with moxidectin was performed in September 2015. The final deworming in the study was performed in May 2016 using pyrantel, with an observed reduction in cyathostomin eggs of 84% at the next monthly sample. The intention of this study was not to evaluate treatment efficacy, but to monitor egg counts on a monthly basis. Thus the reduction in cyathostomin eggs reported here is based on a second sample taken approximately 21–28 days after treatment, and not 10–14 days after treatment as recommended by the guidelines on measuring clinical efficacy of the drug [5]. However, the reduction in cyathostomin eggs and the ERP is not satisfactory according to the latest study performed in Sweden, with an ERP of 5–6 weeks [9]. In summary, the horses were dewormed five times during the study, three times with pyrantel and twice with moxidectin, and the moxidectin treatments were more effective in reducing egg output than pyrantel.

During the study period, the weather conditions were within the expected range in the region [26,27], so the parasite survival observed can be assumed to be representative of the local circumstances. Temperature and moisture are important factors for the development from eggs to infective larvae (stage L3). An experimental study has shown that the development of cyathostomin eggs to L3 occurs at temperatures from +8 to +35 °C in laboratory conditions and takes between 3 and 24 days [28]. A field study in Ukraine (similar winter climate to Sweden) found that strongyle eggs did not develop into L3 when the temperature was below +3 °C, but that L3 which had already developed could survive within feces deposits and act as a reservoir for re-infection of horses at the beginning of the grazing season [29]. 

In our year-round grazing system, the pastures were continually contaminated with parasite eggs for three grazing seasons. There were two peaks of EPG per year, in spring and summer–autumn. We observed a gradual increase in cyathostomin EPG in the horses with every passing year, most likely due to increased numbers of L3 on the pasture, and the horses were re-infected soon after anthelmintic treatment. 

Selective parasite treatment in horses is generally based on individual monitoring of fecal egg counts and treating only individuals that are shedding moderate to high numbers of eggs (>200 EPG) [5]. The aim with this strategy is to reduce total egg contamination of pastures and lower the risk of grazing horses being infected with new parasites. However, it was not possible to determine individual fecal egg counts for all horses in this study, so a modified selective treatment strategy was applied to monitor parasite occurrence. A possible limitation of this approach is that not all horses were sampled on all occasions and therefore the results may be biased by some individuals to a higher extent than others. However, individual horses were sampled at least 14 times during the 2.5-year study, which can be considered quite frequent. To account for this, LSmeans (where missing values are taken into account) were used in presentation of results. 

In addition to infection with cyathostomins, the horses in this study were infected with other gastrointestinal parasites, such as *Parascaris* spp., *A. perfoliata*, and *O. equi*, during the first season (2014/2015). *Parascaris* spp. is considered to be most important parasite of foals, with most individuals developing age-dependent immunity at around one year of age [6]. The horses in our study were 1.5 years of age in winter 2014/2015 and the number of eggs excreted was low (50–150 EPG), most likely due to emerging immunity. After one deworming with fenbendazole, the infection was successfully eradicated. In January–April 2015, infection with *O. equi* spread among the horses, with 10 out of the 12 horses infected. Like *Parascaris* spp., patent *O. equi* infections are reported to be more common in weanlings and young adults [30]. The *O. equi* infection in our horses was effectively treated with fenbendazole in combination with manual cleansing of the perianal area on a weekly basis to decrease transmission of eggs to the surroundings. The *O. equi* infection was the only infection that appeared to cause some discomfort in the horses, as they were observed scratching their tails now and then during this period. One horse also showed mucus excretion from the anus, as described earlier [31]. Eggs of *A. perfoliata* were detected in six out of the 12 horses in May–June 2015. This parasite has an indirect life cycle and is dependent on oribatid mites to complete the cycle [32]. The horses were successfully treated with pyrantel and no re-infection occurred. This suggests that the number of oribatid mites, the intermediate host, was low on the pastures in all enclosures. 

Horses with *B. equi* infestation were found in all groups (A–C), and in total six out of the 12 horses were infested. A large variation in louse counts between individuals housed together has been described in horses [33]. Studies on factors that render individual horses susceptible to lice infestation are scarce. Studies in which sheep were subjected to an equal challenge with *Bovicula ovis* (sheep chewing louse) found large variations in susceptibility between individuals [34]. Interestingly, in that study one-third of the ewes never became infested despite having lice applied on five separate occasions and also being housed together with infested animals. A number of factors, such as season, genotype, age, and body condition, have been suggested to influence susceptibility to lice infestation in sheep [35], with individuals with slow growth and loss of body weight possibly being more susceptible [34,35]. However, in the present study there was no difference in body condition between infested and non-infested horses. The behavioral study showed that scratching behaviors were not more common in infested individuals than in individuals found to be lice-free. In addition, no effects that could be linked to the partial hair loss associated with *B. equi* infestation were observed (e.g., horses were checked daily for shivering). This indicates that an infestation of 5–9 *B. equi* eggs and adults per 4 cm^2^, as found in the present study, induces little or no challenge in affected horses and is therefore probably not a significant health and welfare problem. This knowledge is of relevance for management systems expected to support biological diversity, i.e., minimizing the use of insecticides, in this case anthelmintics. 

As expected, the number of individuals with *Gastrophilus* spp. eggs attached to the hair coat increased in August, but eggs, or at least egg shells, were found until April, i.e., as long as horses still had their winter coat. The possible impact of *Gastrophilus* spp. on the health of the horses in this study is unknown, as the gastric mucosa was not examined. However, while some horses were likely infected with *Gastrophilus* spp., this parasite is generally not associated with any serious health issues [36].

The horses that were taken out of the study due to low body condition did not shed more strongyle eggs than the remaining horses. In wild horses that die from starvation, heavy parasite burdens have been observed [37]. High intestinal parasite loads have also been linked to decreased gut microbial diversity and increased fecal excretion of metabolites [38]. Body condition is reported to be negatively correlated with parasite burden in feral horses [39]. The lower limit of body condition score set for horses to remain in the present study was 4 (scale 1–9), which is still ‘healthy’ condition (corresponding to the fat content of equine athletes [40,41]). The results indicate that at this body condition, horses may still invest energy in resistance to parasites, which may not be the case in horses in poorer body condition [42,43,44]. The level of 4 was chosen because below this horses most likely face challenges in thermoregulation during cold, rainy weather. However, the link between body condition and strongyle egg counts in growing horses needs further investigation.

It is commonly argued that animal density/pasture availability has a strong effect on parasite occurrence [15], indicating that increasing the pasture area would be one possible strategy to limit parasite occurrence. However, in the present study, pasture area and availability did not explain the variation in EPG, with the largest enclosure (2) showing the same EPG counts as the other enclosures. The area of grassland in enclosure 2 was 0.6 ha greater than in the other two enclosures, and it had a significantly greater amount of pasture (1393 ± 114, 957 ± 114, and 741 ± 114 kg dry matter/ha in enclosures 2, 1, and 3, respectively) and significantly greater energy availability (11204 ± 928, 8494 ± 895, and 7351 ± 895 MJ metabolisable energy/ha, respectively) [26]). This implies that an increase of 0.15 ha/horse, or around 125 kg dry matter pasture per horse and month, is not enough to decrease cyathostomin egg excretion and thereby the need for anthelmintic treatment. 

Sweden has been identified as an area where climate conditions may allow free ranging horses [45]. The horse breed used in this study, the Gotlandsruss, was chosen due to its hardy qualities, as it is an ancient breed expected to have the traits required for extensive management. These include low energy requirements and a thick winter coat. Ability to withstand parasite infections may be another desirable trait in free-ranging horses. In this study, cyathostomins were the only endoparasite found in three individual horses, two of which were by the same sire and from the same breeder. There was also a large variation in cyathostomin EPG count between individuals. In addition, only half the horses were infested with chewing louse. Such differences should be taken into account both when selecting animals for year-round grazing systems and in breeding, as immunity to cyathostomins is a heritable trait [42]. If year-round grazing is to be applied on a large scale to increase biodiversity in the cultivated landscape, individual feces sampling and selective treatment may not be feasible. Therefore, animals that excrete low amounts of parasite eggs should be used in these systems, as the contamination of pasture will be reduced. We also made the interesting observation that among the horses in the study, which included five chestnuts and seven bay horses, only bay horses were infested with lice. However, whether *B. equi* has a preference for coat color or linked characteristics needs to be confirmed using a much larger dataset. Interestingly, it has been suggested previously that there could be an association between coat color and tolerance to *Haemonchus contortus* infection in sheep [46].

## 5. Conclusions

We conclude that there was an effect of season on cyathostomin egg excretion by year-round grazing horses in this study, with two peaks observed every year. It was not possible to adapt a pyrantel-only strategy to keep EPG levels <200, but treatment with moxidectin was effective in reducing cyathostomin egg excretion. The study therefore confirms the potential goal conflict between preservation of biological diversity in grazed landscapes and maintaining the health of grazing animals. However, we observed an effect of individual on cyathostomin egg excretion. Therefore, we suggest that when using year-round grazing systems to preserve biological diversity, it is important to select individuals with low strongyle egg excretion (as well as other parasite resistance characteristics, many as yet unidentified), in order to limit the number of anthelmintic treatments required. Further studies are required to determine how pasture area and characteristics affect parasite occurrence.

## Figures and Tables

**Table 1:** The experimental design, which comprised a Latin square with three periods, three enclosures, and three groups of horses (A, B, and C).

Period	Enclosure 1	Enclosure 2	Enclosure 3
May 2014–May 2015	A	B	C
May 2015–May 2016	B	C	A
May 2016–September 2016	C	A	B

**Table 2:** Parasite occurrence in fresh feces samples collected monthly from horses 1–12 during the 2.5-year study period. Horses were tracked until a minimum of two horses/enclosure defecated, and n equals the total number of samples for each horse.

Horse	n	Strongyle Eggs	*Parascaris* spp.	*A. perfoliata*	*S. vulgaris*	*O. equi*	*B. equi*	Botfly Eggs ^‡^
1	21	x	x	x		x	x	x
2 ^‡‡^	14	x				x	x	x
3	21	x	x	x		x		x
4	21	x		x		x		x
5	15	x	x			x		x
6	18	x				x	x	x
7	16	x					x	x
8	17	x		x		x	x	x
9	14	x				x		x
10 ^‡‡‡^	18	x						x
11	19	x		x		x		x
12	20	x		x		x	x	x

^‡^*Gasterophilus* spp. eggs found in coat. ^‡‡^ Horse 2 was excluded from the study in March 2016 because of an injury. ^‡‡‡^ Horse 10 was not sampled for *Oxyuris equi*.

**Table 3:** Strongyle eggs per gram (EPG) in feces samples taken monthly from 12 horses kept in a year-round grazing regime between May 2014 and September 2016. LSmeans ± SEM. Superscript letters (a, b, c) indicate a significant difference between years, seasons, enclosures and horse groups.

Items	Year			*p*-Value
	1	2	3	
EPG	101 ± 24 ^a^	267 ± 37 ^b^	415 ± 67 ^c^	<0.0001
		**Season**		
	**Spring**	**Summer–Autumn**	**Winter**	
EPG	141 ± 28 ^a^	725 ± 75 ^b^	110 ± 32 ^a^	<0.0001
		**Enclosure**		
	**1**	**2**	**3**	
EPG	246 ± 41	184 ± 33	247 ± 41	n.s.
	**Horse group**			
	**A**	**B**	**C**	
EPG	192 ± 35 ^a^	349 ± 55 ^b^	216 ± 38 ^a^	<0.01
